# Is the anterior drawer test still valuable for diagnosing mechanical ankle instability in clinical practice and research?

**DOI:** 10.3389/fbioe.2025.1664779

**Published:** 2025-09-09

**Authors:** Lijiang Luan, Wenxuan Ji, Charlotte Ganderton, Joshua Farragher, Evangelos Pappas, Roger David Adams, Shasha Wang, Jia Han

**Affiliations:** ^1^ Jiangwan Hospital of Hongkou District, Shanghai, China; ^2^ School of Physical Education and Health, Sanming University, Sanming, China; ^3^ College of Rehabilitation Sciences, Shanghai University of Medicine and Health Sciences, Shanghai, China; ^4^ School of Health and Biomedical Sciences, RMIT University, Melbourne, VIC, Australia; ^5^ Department of Nursing and Allied Health, Swinburne University of Technology, Melbourne, VIC, Australia; ^6^ Research Institute for Sport and Exercise, University of Canberra, Canberra, ACT, Australia; ^7^ School of Health Sciences, The University of Sydney, Sydney, NSW, Australia; ^8^ Tongji University, Shanghai, China

**Keywords:** anterior drawer test, mechanical ankle instability, reliability, validity, minimal detectable change

## Abstract

The anterior drawer test (ADT) has been widely used in assessing mechanical ankle instability (MAI), yet its applicability has been questioned. This study aims to clarify the clinical value of the ADT. Five databases were searched in January 2025. Studies investigating the properties of the ADT were included. Data on reliability, validity, diagnostic accuracy, and responsiveness were extracted. A total of 424 studies were screened, and 45 studies were included. The ADT generally demonstrated good to excellent intra-rater reliability, but relatively poor inter-rater reliability. Criterion validity was supported by moderate to strong correlations with imaging and anatomical measurements. The diagnostic accuracy of ADT and its variants, such as the anterolateral and reverse ADTs, showed to be generally favorable. Inconsistencies in responsiveness across studies highlighted the need for population-specific classifications and the urgent establishment of corresponding measurement standards. The manual ADT exhibited a minimal detectable change value of 1.995 mm for intra-rater reliability. Overall, the ADT provides valuable insights for diagnosing MAI, but its inter-rater reliability and accuracy may be influenced by examiners’ clinical experience and testing methods. Standardized protocols and advanced grading systems are needed to minimize inter-rater variability and enhance its result consistency, precision, and clinical utility.

## Introduction

The stability of the ankle joint plays a crucial role in maintaining balance and lower limb movement functions (Brown et al., 2015). While neuromuscular control is vital, ankle stability significantly depends on the structural integrity of the joint itself (Kirby et al., 2005). Ankle sprains can lead to varying degrees of ligamentous damage, potentially resulting in chronic ankle instability (Kerkhoffs et al., 2012; Lindstrand and Mortensson, 1977). Accurate diagnosis of the ligamentous integrity and mechanical stability is crucial for formulating management strategies, developing training regimens, and assessing intervention effectiveness (Vuurberg et al., 2018).

Clinically, ankle stability is typically assessed using three primary methods. The first involves imaging modalities, often considered the gold standard, such as ultrasound, magnetic resonance imaging, and stress radiography (Kerkhoffs et al., 2012; Vuurberg et al., 2018). These techniques provide relatively accurate assessments of joint laxity or ligamentous structural integrity, but they are often time-consuming and costly (Kirby et al., 2005; Chang et al., 2021; Park, 2021). The second method is direct observation via surgery, such as arthroscopy; although this may be accurate, it is invasive and expensive (Vuurberg et al., 2018; Chang et al., 2021). The third and more commonly utilized approach is manual testing, which provides a timely and economical means of assessing ankle stability (Sun et al., 2018; Netterstrom-Wedin et al., 2022; Beynon et al., 2022).

Despite the convenience of manual testing, concerns about its reliability, validity, and diagnostic accuracy persist (Park, 2021; Netterstrom-Wedin et al., 2022; Beynon et al., 2022). These factors are critical for evaluating a diagnostic tool’s effectiveness and align closely with the COSMIN guidelines, which provide core standards for assessing measurement tool quality (Mokkink et al., 2010). By investigating these aspects, one can gain a comprehensive understanding of the strengths and weaknesses of the ADT, offering clearer insights into its results and providing valuable guidance for health professionals when selecting diagnostic tools (Netterstrom-Wedin et al., 2022; Beynon et al., 2022; Mokkink et al., 2010). A diagnostic tool built on solid retest properties ensures reliable and valid measurements, supporting its use in clinical practice and research (Vuurberg et al., 2018; Chang et al., 2021).

Despite its prevalence, a systematic evaluation of the ADT remains lacking, and debates about its efficacy and accuracy persist (Netterstrom-Wedin et al., 2022). Therefore, this review aims to investigate the ADT, providing evidence-based guidance on its applicability as a diagnostic tool for assessing ankle instability.

## Methods

This study has been registered in the International Prospective Register of Systematic Reviews (PROSPERO) database (registration number: CRD42024585466).

### Search strategy

A systematic search was conducted in January 2025 using PubMed, Embase, Cochrane Library, Web of Science, and EBSCO databases to identify studies involving the use of ADT for the ankle joint. The search strategy focused on the terms ‘ankle,’ ‘ADT,’ and characteristics related to the reliability and retest evaluation of the drawer test methods (Supplementary Material S1A). Additionally, studies related to clinical examination of the ankle joint were screened, and relevant references were reviewed to identify additional studies for inclusion.

### Study selection

The studies obtained from the systematic search and those included in relevant reviews were screened. The titles and abstracts of identified studies were initially screened to exclude ineligible studies. After the initial screening, eligibility was further assessed through full-text review based on the following inclusion criteria: (1) the ADT was performed on the ankle joint; (2) the evaluation results of the ADT were reported; (3) at least one psychometric property of the ADT was reported. Exclusion criteria: animal experiments, simulators or prosthetics, non-experimental detection surveys, case reports, reviews, and clinical trial registrations.

### Quality assessment

The quality of the studies that met the inclusion criteria was assessed using the Critical Appraisal Tool (CAT) (Brink and Louw, 2012), which is based on the QUADAS and QAREL (Whiting et al., 2003; Lucas et al., 2010). The CAT is scientifically robust, and its details are provided in Supplementary Material S1B.

### Outcome measures

This study investigated the reliability and validity of the ADT, which are key components in the evaluation of clinical diagnostic tools (Beynon et al., 2022). Assessing the reliability and validity of the ADT is crucial for ensuring accurate test results and robust statistical conclusions (Beynon et al., 2022). Additionally, diagnostic accuracy and responsiveness were also key outcomes in this study, as the evaluation of these two aspects can measure the practical utility of the anterior drawer test, thereby reflecting its applicability (Sman et al., 2013). Specifically, regarding reliability, the ADT does not involve multiple items, so the outcome of internal consistency was excluded, with the focus mainly on test-retest reliability (intra/inter-rater). Regarding validity, this study did not delve into content validity, based on subjective evaluations; rather, it primarily analyzed construct validity and criterion validity. A focus was placed on specificity and sensitivity as they sufficiently reflect diagnostic accuracy; indicators such as likelihood ratio, predictive value, and accuracy, were not elaborated on, which can be derived through calculations (Netterstrom-Wedin et al., 2022; Sman et al., 2013). Regarding responsiveness, considering the need to provide some reference value for clinical evaluation, this study primarily reviewed the displacement results of the ADT.

### Data extraction

Data on eligible studies were extracted, including demographic information and diagnostic conditions. To focus on the measurement effectiveness of the ADT, this study only considered participants who had undergone the ADT. Examiner experience performing the ADT, implementation measures, testing modes, and the evaluation methods used were summarized. Further, specific data on reliability, validity, diagnostic accuracy, responsiveness are provided in Supplementary Material S1C.

### Exploration of the minimal detectable change

The Minimal Detectable Change (MDC) represents the smallest measurable difference that exceeds the measurement error of an assessment tool, indicating true clinical change rather than random error or variability (Vuurberg et al., 2018; Netterstrom-Wedin et al., 2022). MDC is calculated using the Standard Error of Measurement (SEM), quantifying the tool’s inherent measurement error. MDC reflects the tool’s sensitivity in detecting clinically meaningful changes, serving as a crucial reference in both clinical practice and research (Sman et al., 2013; Phisitkul et al., 2009; Croy et al., 2013). Therefore, exploring the MDC values in ADT is of great significance and key guiding role, and the specific methods adopted in this study are described in Supplementary Material S1D.

## Results

### Literature search and screening

**FIGURE 1:**
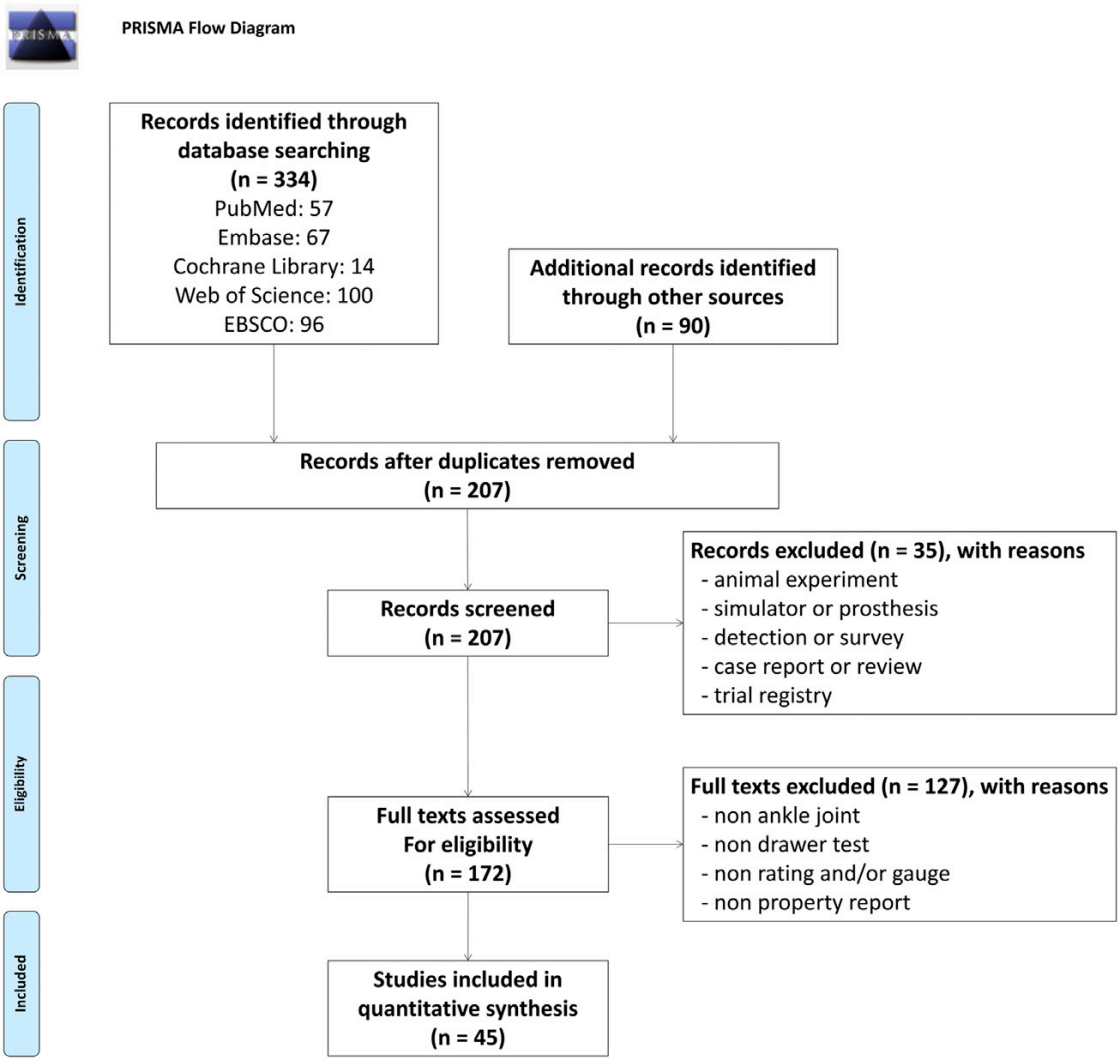
Flowchart of Literature Search and Screening.

A total of 424 studies were identified through database searches and references from related reviews. After removing duplicate studies, the titles or abstracts of 207 studies were screened. Subsequently, 172 full-text studies were reviewed, with 127 excluded for not addressing the ankle ADT or relevant properties. Ultimately, 45 studies (3,474 participants) met the inclusion criteria for this review (Phisitkul et al., 2009; Croy et al., 2013; Gomes et al., 2018; Parasher et al., 2012; Vaseenon et al., 2012; George et al., 2020; Hosseinian et al., 2022; Großterlinden et al., 2016; Wilkin et al., 2012; van Dijk et al., 1996a; Li et al., 2020; Wiebking et al., 2015; Spahn, 2004; Wenning et al., 2021; van Dijk et al., 1996b; Chen et al., 2022; Lin et al., 2013; Raatikainen et al., 1992; van den Hoogenband et al., 1978; Prins, 1978; Lindstrand, 1976; Funder et al., 1982; Cho et al., 2016; Chandnani et al., 1994; Ahovuo et al., 1988; Rijke and Vierhout, 1990; Blanshard et al., 1986; Johannsen, 1978; Beumer et al., 2002; Yokoe et al., 2023; Yokoe et al., 2022; Azni et al., 2020; Chen et al., 2023; Iwata et al., 2023; Sillevis et al., 2022; Song et al., 2021; Murahashi et al., 2023; Teramoto et al., 2021; Kataoka et al., 2022; Yokoe et al., 2021; Kawabata et al., 2023; Docherty and Rybak-Webb, 2009; Saengsin et al., 2022; Gulick, 2024; Iwata et al., 2024). The selection process and reasons for exclusion are illustrated in Figure 1.

### Quality assessment of included studies

Most included studies that assessed the reliability and/or validity of the ankle ADT demonstrated acceptable methodological rigor; however, several limitations were identified. The detailed results of the quality assessment are summarized in Supplementary Material S1E.

### Characteristics of included studies

Among the eligible studies, most included participants with an ankle injury. The qualifications of the examiners performing the ADT varied. Most studies focused on assessing the anterior talofibular ligament (ATFL), with some studies also including the calcaneofibular ligament (CFL). The drawer test methods included traditional anterior drawer test (TADT), anterolateral drawer test (ALDT), reverse anterior drawer test (RADT), reverse anterolateral drawer test (RALDT), and instrumented anterior drawer test (IADT). The most common evaluation method was the subjective judgment by the examiner, with some studies using imaging as a reference for rating. Diagnostic accuracy studies primarily used imaging as the reference standard. The specific content is detailed in Supplementary Material S1F.

### Properties of ADTs

Despite inconsistencies in study populations, examiners, and experimental conditions, the reliability of various ADT modes was generally satisfactory for intra-rater assessment, as reflected by ICC and Kappa values (Parasher et al., 2012; Kataoka et al., 2022). In contrast, most inter-rater reliability results were poor to moderate, only one or two studies reached high values (Chen et al., 2022; Saengsin et al., 2022). Factors like examiner experience can affect inter-rater reliability, highlighting the need for careful assessment protocols. Three studies reported lower reliability (Wilkin et al., 2012; Li et al., 2020; Beumer et al., 2002), primarily due to small sample sizes and atypical testing positions (e.g., supine). Overall, the ADT and its variants (ALDT, RADT, IADT) generally maintain moderate to excellent reliability.

In terms of criterion validity, ADT generally showed moderate to strong positive correlation with imaging modalities such as ultrasound, magnetic resonance imaging, and stress radiography (Gomes et al., 2018; Saengsin et al., 2022). However, research on construct validity remains limited, with three studies showing low correlation between ADT and certain scales such as CAIT, VAS, and Beighton (Wilkin et al., 2012; Spahn, 2004; Yokoe et al., 2022), while two studies indicating high correlation between ADT and CAIT as well as Beighton score (Wenning et al., 2021; Song et al., 2021). Further exploration of the subjective assessment correlation is necessary due to this contradiction.

ADT’s sensitivity and specificity varied across studies depending on parameters associated with the condition of ankle instability and examiner experience. Good specificity was observed in patients with acute supination trauma, while sensitivity was often poor in those reporting chronic ankle instability (Gomes et al., 2018; Funder et al., 1982). Parameters such as duration of instability and ligament injury degree impact diagnostic accuracy. One study found that senior and junior raters showed 80% and 40% sensitivity for the ADT, respectively (Murahashi et al., 2023).

The responsiveness of ADT varied across studies, with significant differences in displacement values between patient groups. For instance, one study reported a mean displacement of 4.5 mm for senior examiners vs. 3.26 mm for junior examiners (Murahashi et al., 2023). Specifically, mean displacement of 3.2 mm in injured ATFL and 0.6 mm in intact ATFL were observed (Iwata et al., 2024). Significant displacement differences were noted in patients with CAI or ligament injury, yet within-group variability remained high (Saengsin et al., 2022).

Detailed data above are presented in Supplementary Material S2A–D, respectively.

### The minimal detectable change of the ADT

Three studies reported both ICC and SEM values for reliability outcomes (Parasher et al., 2012; Wilkin et al., 2012; Docherty and Rybak-Webb, 2009). Additionally, one study provided ICC (intra) and standard deviation (SD) for each rater’s measurements assessing intra-rater reliability (Kataoka et al., 2022). Calculations indicated that manual ADT testing exhibited a maximal MDC of 1.995 mm for intra-rater reliability, while the MDC for instrumented ADT testing was higher, at 6.153 mm. Furthermore, discrepancies between raters in ADT application showed a maximal MDC of 6.291 mm or 4.684 units on an 8-point Likert scale. Detailed results are presented in Supplementary Material S2E.

## Discussion

While certain limitations exist, such as inter-rater reliability issues and subjective grading, the ADT remains a practical, cost-effective, and widely accepted tool in clinical practice. By synthesizing insights from reliability and validity data, sensitivity and specificity reports, and MDC analyses, this discussion underscores the ADT’s continued relevance and identifies areas for refinement, including standardized protocols and enhanced grading systems.

### The properties of the ADT

As noted, the reliability of the ADT is generally high, with most studies reporting moderate to excellent intra-rater and inter-rater reliability. However, several studies highlight that test reliability is significantly affected by factors such as examiner experience, test variations, and subject positioning. Specifically, intra-rater reliability is generally high, particularly when the same examiner conducts the test multiple times. However, differences are evident when examining the same test performed by different raters, indicating that examiner experience can impact on reliability. Additionally, ADT’s reliability was lower when using multiple variations of the test (e.g., ALDT, RADT) compared to the traditional approach (Li et al., 2020; Beumer et al., 2002), suggesting that more complex testing variations may increase error and variability. Therefore, proper training and uniform test protocols are essential to minimize discrepancies and enhance the consistency of results.

The validity of the ADT is primarily assessed by comparing its results with imaging and surgical findings. Most studies demonstrate moderate to strong criterion validity through correlations with imaging modalities, confirming that ADT results align reasonably well with imaging assessments. However, construct validity remains less well-explored, and the limited evidence mainly concerns rating scales. For example, the Beighton scale, commonly used to evaluate generalized joint hypermobility by assessing the range of motion in the little finger, thumb, elbow, knee and trunk, which is a 9-point clinical scoring system (Yokoe et al., 2022; Song et al., 2021). In this review, a correlation of r = 0.719 between Beighton scores and ADT was found (Song et al., 2021), indicating a strong relationship in some populations, while Beighton scores did not demonstrate significant correlations with ADT results in the other two studies (Yokoe et al., 2023; Yokoe et al., 2022). This discrepancy highlights that ankle instability as measured by ADT may not be fully captured by generalized joint laxity scores, reinforcing the need for a more comprehensive assessment method that incorporates both global and local assessment.

The diagnostic accuracy of ADT is generally favorable but varies based on factors like examiner experience, test timing, and patient characteristics. For instance, two studies revealed stark differences between novice and experienced raters (Li et al., 2020; Murahashi et al., 2023), highlighting how examiner proficiency impacts test outcomes. As variations of the drawer test, such as ALDT, RADT, and IADT, experience less impact from examiner experience, potentially making them better suited for novices. Test timing also influences diagnostic outcomes; certain studies found that sensitivity and specificity differed significantly between 48 h and 5 days after injury (van Dijk et al., 1996a; van Dijk et al., 1996b). These findings underscore the importance of timing in diagnostic accuracy, suggesting ADT is more reliable after the acute inflammatory phase has subsided. Patient characteristics, like injury severity and the acute vs. chronic nature of the instability, can also affect diagnostic accuracy. For instance, two studies demonstrated lower accuracy in patients with chronic syndesmotic ruptures (Johannsen, 1978; Beumer et al., 2002), likely due to injury complexity. Additionally, higher diagnostic accuracy was found in patients with severe injuries (Raatikainen et al., 1992), suggesting that ADT may be more effective in detecting more pronounced mechanical instability. Therefore, ADT’s diagnostic accuracy is highly dependent on contextual factors such as examiner expertise, timing of conduction in relation to injury and injury severity.

The responsiveness of ADT varies across studies, likely influenced by examiner experience and patient demographics, including injury severity and time since injury. Differences in displacement between senior and junior examiners were noted (Murahashi et al., 2023), indicating that examiner proficiency impacts test responsiveness. A study observed a difference between injured and intact ATFL (Iwata et al., 2024), suggesting acute injuries produce more pronounced displacement, whereas chronic cases may show less sensitivity to ADT. This indicates that the measurement of ADT is likely more effective for acute injury than for chronic condition. Displacement differences were also noted across symptomatic conditions; chronic injuries and multi-ligament tears (ATFL + CFL) showed larger displacements compared to isolated ATFL injuries (Saengsin et al., 2022). These differences indicate that ADT’s responsiveness is influenced by both examiner experience and injury severity, which affects ligamentous stability and displacement readings. Thus, establishing population-specific MDC and MCID values is essential for improving ADT’s responsiveness in clinical settings.

### The minimal detectable change for the ADT and additional considerations

In this study, the MDC for the ADT was explored as an essential measure of the smallest clinically relevant change that exceeds measurement error. Based on the MDC values and findings across studies (Supplementary Material S2E), we can derive several key considerations for improving the precision and clinical applicability of the ADT.

When performing ADT, even experienced raters may show considerable error between their results. This indicates variability exists between raters, even among experienced examiners. Therefore, when multiple raters are involved in ADT assessments, it is crucial to be cautious with the analysis of the results, as discrepancies can arise.

Manual ADT testing may demonstrate smaller errors compared to instrumented ADT. This could be due to the inherent stability of manual testing, where the rater’s movements are typically controlled until no further motion is observed (Parasher et al., 2012; Wilkin et al., 2012; Docherty and Rybak-Webb, 2009). In contrast, instrumented ADT often operates with a fixed force, such as 80 N, 120 N, or 150 N (Chen et al., 2022; Kataoka et al., 2022). However, due to the elastic nature of joint tissues, the sliding distance in instrumented ADT may vary as soft tissues adapt, leading to larger measurement errors (Wiebking et al., 2015; Chen et al., 2022; Murahashi et al., 2023). On the other hand, in manual ADT, the same maximum sliding distance is more consistently achieved with each assessment, resulting in smaller error margins (Parasher et al., 2012; Wilkin et al., 2012).

Using imaging devices, such as fluoroscopic images, results in smaller measurement errors and greater precision compared to tools like goniometers or arthrometers (Parasher et al., 2012; Saengsin et al., 2022). Although imaging provides a clearer and more accurate method of measuring, it is a more costly and time-consuming approach (Parasher et al., 2012; Sillevis et al., 2022). This trade-off must be considered when determining the most appropriate measurement method for clinical or research purposes.

The common grading system for ADT evaluations typically uses three levels (1, 2, 3), which are rather broad and lack the precision needed to distinguish subtle differences (Parasher et al., 2012; Vaseenon et al., 2012). For instance, the difference between level 2 and level 3 may not be significant enough to exceed the MDC, leading to potential misclassification. Additionally, using just three to four grading levels makes it difficult to effectively and accurately assess measurement errors. It is suggested that future ADT evaluations use a finer scale, such as an 8-point Likert scale, to improve precision in detecting measurement errors and enhance the validity of MDC estimation.

### Recommendations for improving the ADT

Based on the characteristics, current applications, and identified limitations of the ADT discussed above, several recommendations for improvement are proposed to enhance its clinical utility.

While instrumented and imaging-based ADT methods offer measurable advancements in precision, they are not without limitations. Instrumented drawer tests apply fixed forces, which fail to account for individual differences such as foot length, body weight, or joint elasticity, which makes it challenging to achieve reliable results in diverse populations. Similarly, imaging-based methods, while providing precise displacement values, are often impractical in clinical settings due to their cost, inefficiency, and unclear clinical significance of the measurements.

In contrast, manual drawer testing remains a highly adaptable and patient-centered approach. Skilled therapists can adjust force application based on the patient’s individual characteristics, minimizing variability caused by population differences. Furthermore, manual tests are quick, cost-effective, and well-suited to clinical practice. These qualities underscore the importance of continuing to rely on manual drawer tests while addressing their inherent limitations.

Despite its practicality, manual drawer testing has shortcomings. Variability among raters, as highlighted by differences in intra-rater and inter-rater reliability, presents a significant challenge. MDC intra-rater values tend to be lower and more consistent compared to inter-rater MDC values, reflecting the added variability when multiple raters are involved. Some studies demonstrated noticeable disparities in reliability across different raters (Parasher et al., 2012; Wilkin et al., 2012).

To enhance the consistency of manual testing, several recommendations are proposed: (a) Standardized protocols: Establish comprehensive and highly detailed operating procedures for ADT, including standardized verbal instructions and consistent force application techniques; also, current studies provide limited reporting on the position of the ankle in testing (dorsiflexion, neutral, plantarflexion), making deeply comparisons difficult. However, different testing positions may exert varying influences on the ATF and CF ligaments, highlighting the need for future research to standardize the specific ankle position during ADT implementation. (b) Training and certification: Implement rigorous training programs to ensure that examiners achieve a high level of proficiency before clinical application. Practical assessments and certification processes should be developed to evaluate the competency of testers, aligning their skills to a consistent standard. (c) Continuous monitoring: Encourage single-rater assessments for individual patients whenever feasible, as this minimizes inter-rater variability and strengthens reliability over the course of treatment. (d) Transition to a more granular grading scale, such as a 0–10 Likert scale or a modified VAS scale. These systems can offer enhanced precision, reflecting a continuum from “no instability” to “complete instability”. Expanding the ADT scoring system from the traditional 0–3/4 grades to a broader 0–8 or 0–10 scale may offer notable advantages. First, it increases the sensitivity of the assessment by allowing finer gradations of anterior talar translation, thereby capturing subtle variations in ankle laxity that might be overlooked with a narrower scale. Second, a broader scoring range enhances clinical and research applicability by improving the ability to discriminate between patients with different levels of instability, reducing ceiling effects, and enabling more precise comparisons of therapeutic outcomes. (e) Develop population-specific MDC and MCID thresholds to better guide clinical decision-making for subgroups like patients with structural ankle instability. This refinement would enhance the accuracy and clinical utility of manual ADT, facilitating more precise diagnoses and targeted interventions.

These recommendations aim to bridge the gap between manual and advanced ADT methods, ensuring that manual drawer testing remains a cornerstone of ankle instability assessment while evolving to meet the demands of modern clinical and research practices. Study limitations are presented in Supplementary Material S2F.
