# Supplementary material for: Is the anterior drawer test still valuable for diagnosing mechanical ankle instability in clinical practice and research?
Source: Front Bioeng Biotechnol. 2025 Sep 9;13:1664779. doi: 10.3389/fbioe.2025.1664779 (PMC12454323; doi:10.3389/fbioe.2025.1664779)
Supplement: Supplementary file 1 [file Table1.docx]

**(A) Search Strategy**

[Title/Abstract] (((chronic OR functional OR mechanical) AND (ankle instability OR instability, ankle OR instable ankle OR unstable ankle)) OR ((recurrent ankle OR ankle recurrent) AND (sprain* OR injur*)) OR ((ankle ligament* OR ankle joint*) AND (laxity OR relaxation)))

AND

[Title/Abstract] ((anter* drawer) AND (test* OR diagnos* OR exam* OR measure* OR assess* OR evalu* OR tool* OR screen*))

AND

[Title/Abstract] (reliab* OR valid* OR reproduc* OR propert* OR adaptation OR test-retest OR correlate* OR repeated measure OR intra-class coefficient OR ICC OR cronbach OR kappa OR association OR responsiveness OR MDC OR minimal detectable change OR MCID OR minimal clinical important difference OR minimal clinical important change OR internal consistency OR content OR construct OR convergent OR divergent OR sensitivity OR specificity)

There were no restrictions on language or publication year.

**(B) Critical Appraisal Tool (CAT)**

This tool was developed by Dr. Yolandi and colleagues from the University of South Australia, following the Delphi method. It is based on two quality assessment tools: the Quality Assessment of Diagnostic Accuracy Studies (QUADAS), developed by Whiting and colleagues at the University of York, UK, in 2003, and the Quality Appraisal of Diagnostic Reliability Studies (QAREL), developed by Lucas and colleagues in 2010. The QUADAS tool is a widely recommended tool for assessing the quality of diagnostic accuracy studies in systematic reviews and is also adopted by the Cochrane Collaboration’s diagnostic test systematic review methodology group. The QAREL tool is specifically designed for the quality assessment of diagnostic reliability studies and has been widely used and recommended. Therefore, the CAT is based on reliable sources and is scientifically robust.

The CAT is a rigorously evaluated and validated standard for assessing the quality of tests. Its development underwent five stages: forming the conceptual framework, identifying key elements, creating an item pool, testing face validity (using the Delphi method), and finalizing the tool. It consists of 13 items, with 5 items (1, 2, 10, 12, 13) used to assess both reliability and validity, 4 items (3, 7, 9, 11) addressing validity only, and 4 items (4, 5, 6, 8) focusing on reliability solely. Each item is rated as “yes”, “no”, or “not applicable”. When using the CAT to assess studies involving only reliability, the items referring to only validity studies should be rated as “not applicable” and vice versa.

**(C) Extracting data of reliability, validity, accuracy, responsiveness**

Reliability reports from the studies were extracted, including but not limited to the following data: Cohen’s Kappa (κ) (< 0: disagreement (worse than random), 0-0.20: slight agreement, 0.21-0.40: fair agreement, 0.41-0.60: moderate agreement, 0.61-0.80: substantial agreement, 0.81-1.00: almost perfect agreement); Intraclass Correlation Coefficient (ICC) (< 0.50: poor reliability, 0.50-0.75: moderate reliability, 0.75-0.90: good reliability, > 0.90: excellent reliability); Standard Error of Measurement (SEM) (the smaller the value, the smaller the measurement error, and the higher the measurement precision); Limits of Agreement (LOA) (the narrower the range between the upper and lower limits, the better the agreement between the measurement methods).

In terms of validity, common evaluation methods include Pearson Correlation Coefficient (PCC/r) (-1: perfect negative correlation, -0.7 to -0.9: strong negative correlation, -0.4 to -0.6: moderate negative correlation, -0.1 to -0.3: weak negative correlation, 0: no correlation, 0.1 to 0.3: weak positive correlation, 0.4 to 0.6: moderate positive correlation, 0.7 to 0.9: strong positive correlation, 1: perfect positive correlation); Spearman's Rank Correlation Coefficient (ρ/rho) (the value range and evaluation standards are the same as those for PCC); Chi-Square Test (χ²) (the larger the value, the greater the deviation between the observed and expected values), etc.

Regarding diagnostic accuracy, for the sake of statistical comparison and brevity, the study did not elaborate on Receiver Operating Characteristic (ROC) curves and Area Under the Curve (AUC) graphs but directly recorded sensitivity data (close to 1 indicates the test effectively identifies individuals with the disease; 0.90-1.00: high sensitivity, 0.70-0.89: moderate sensitivity, 0.50-0.69: low sensitivity, < 0.50: very low sensitivity; close to 0 indicates the test is prone to missing diagnoses) and specificity data (close to 1 indicates the test effectively identifies individuals without the disease; 0.90-1.00: high specificity, 0.70-0.89: moderate specificity, 0.50-0.69: low specificity, < 0.50: very low specificity; close to 0 indicates the test is prone to false positives).

For responsiveness, as the Minimal Clinically Important Difference (MCID) and Minimal Detectable Change (MDC) of the anterior drawer test have not yet been established, only effect size or measurement change data were extracted.

**(D) Exploring the MDC values in ADT**

In this study, for included studies that reported the intraclass correlation coefficient (ICC) and SEM as part of reliability testing, the MDC was calculated directly using the formula: $MDC=SEM\times\sqrt{2}\times Z$ (95% confidence interval: $Z=1.96$). This approach enables the relatively accurate determination of MDC values, facilitating comparative analysis of outcomes obtained through assessments.

Furthermore, in cases where MDC was derived from the intra-rater reliability, it primarily reflects the measurement error attributable to the ADT itself when used by the same rater on different occasions. This type of MDC highlights the intrinsic properties of the ADT in reducing variability. If a study reported a unified intra-rater ICC and provided individual raters’ standard deviations (SDs), the SEM for each rater was calculated using: $SEM=SD\times\sqrt{1-{ICC}_{intra}}$ . This allowed for the indirect calculation of MDC for each rater. Admittedly, while using a unified ${ICC}_{intra}$ for all raters introduces limitations due to its lack of specificity to individual raters, it still provides a meaningful approximation when the evaluations of all raters are relatively consistent. However, if studies only reported ${ICC}_{intra}$ values without corresponding SDs, neither SEM nor MDC could be calculated.

For MDC derived from the inter-rater reliability, it captures the variability arising from different raters performing the ADT. While this type of MDC provides less insight into the ADT’s intrinsic accuracy, it may reveal phenomena related to the consistency of clinical assessments across raters. Noteworthy, it cannot be indirectly calculated using individual rater SDs because the inter-rater SEM represents the collective variance among raters, not individual contributions. Therefore, this MDC can only be calculated directly when both ${ICC}_{inter}$ and SEM are explicitly reported in the study.

When studies lacked sufficient data to calculate MDC values or reported these inconsistently, such limitations were noted, and their potential impact on the interpretation of findings was considered in the synthesis.

**(E) CAT Quality Assessment of Included Studies**

| **Study** | **1** | **2** | **3** | **4** | **5** | **6** | **7** | **8** | **9** | **10** | **11** | **12** | **13** |
| --- | --- | --- | --- | --- | --- | --- | --- | --- | --- | --- | --- | --- | --- |
| Ahovuo 1988 | Y | N | Y | N/A | N/A | N | Y | N/A | Y | Y | Y | N | Y |
| Azni 2020 | Y | N | Y | N/A | N/A | N/A | Y | N/A | Y | Y | Y | Y | Y |
| Beumer 2002 | Y | Y | Y | Y | Y | Y | Y | Y | Y | Y | Y | N | Y |
| Blanshard 1986 | Y | N | Y | N/A | N/A | N/A | Y | N/A | Y | Y | Y | Y | Y |
| Chandnani 1994 | Y | Y | Y | N/A | N/A | N/A | Y | N/A | Y | Y | Y | N | Y |
| Chen 2022 | Y | N | Y | Y | Y | N | Y | Y | Y | Y | Y | Y | Y |
| Chen 2023 | Y | Y | Y | N/A | N/A | N/A | Y | N/A | Y | Y | Y | Y | Y |
| Cho 2016 | Y | N | Y | N/A | N/A | N/A | Y | N/A | Y | Y | Y | N | Y |
| Croy 2013 | Y | Y | Y | N/A | N/A | N/A | Y | N/A | Y | Y | Y | Y | Y |
| Docherty 2009 | Y | Y | N/A | Y | Y | Y | N/A | Y | N/A | Y | N/A | N | Y |
| Funder 1982 | Y | N | Y | N/A | N/A | N | Y | N/A | Y | Y | Y | N | Y |
| George 2020 | Y | Y | Y | N/A | N/A | N/A | Y | N/A | Y | Y | Y | N | Y |
| Gomes 2018 | Y | Y | Y | N/A | N/A | N/A | Y | N/A | Y | Y | Y | Y | Y |
| Großterlinden 2016 | Y | Y | Y | Y | N/A | N/A | Y | N/A | Y | Y | Y | Y | Y |
| Gulick 2024 | Y | N | Y | N/A | N/A | N/A | Y | N/A | Y | Y | Y | N | Y |
| Hosseinian 2021 | Y | Y | Y | Y | N/A | N/A | Y | N/A | Y | Y | Y | Y | Y |
| Iwata 2023 | Y | Y | Y | N/A | N/A | Y | Y | N/A | Y | Y | Y | N | Y |
| Iwata 2024 | Y | Y | Y | N/A | N/A | Y | Y | N/A | Y | Y | Y | N | Y |
| Johannsen 1978 | Y | Y | Y | N/A | N/A | N | Y | N/A | Y | Y | Y | N | Y |
| Kataoka 2022 | Y | Y | Y | Y | Y | N | Y | Y | Y | Y | Y | N | Y |
| Kawabata 2023 | Y | Y | Y | Y | Y | N | Y | Y | Y | Y | Y | N | Y |
| Li 2020 | Y | Y | Y | Y | N/A | N/A | Y | N/A | Y | Y | Y | N | Y |
| Lin 2013 | Y | N | Y | N/A | Y | N | Y | N | Y | Y | Y | N | Y |
| § Lindstrand 1976 | - | - | - | - | - | - | - | - | - | - | - | - | - |
| Murahashi 2023 | Y | Y | Y | Y | N/A | N/A | Y | N/A | Y | Y | Y | Y | Y |
| Parasher 2012 | Y | N | N/A | Y | Y | Y | N/A | Y | N/A | Y | N/A | N | Y |
| Phisitkul 2009 | N | Y | Y | Y | N/A | N | N/A | N/A | Y | Y | Y | N | Y |
| § Prins 1978 | - | - | - | - | - | - | - | - | - | - | - | - | - |
| Raatikainen 1992 | Y | N | Y | N/A | N/A | N/A | Y | N/A | Y | Y | Y | Y | Y |
| Rijke 1990 | Y | N | Y | N/A | N/A | N/A | Y | N/A | Y | Y | Y | N | Y |
| Saengsin 2022 | Y | N | Y | Y | Y | N | N/A | Y | Y | Y | Y | N | Y |
| Sillevis 2022 | Y | Y | Y | N/A | N/A | N/A | Y | N/A | Y | Y | Y | N | Y |
| Song 2021 | Y | Y | Y | Y | Y | N | Y | N | Y | Y | Y | N | Y |
| Spahn 2004 | Y | N | Y | N/A | N/A | N/A | Y | N/A | Y | Y | Y | N | Y |
| Teramoto 2021 | Y | Y | Y | Y | Y | N | Y | N | Y | Y | Y | N | Y |
| van Dijk 1996 | Y | Y | Y | N/A | N/A | N/A | Y | N/A | Y | Y | Y | Y | Y |
| van Dijk 1996 (b) | Y | Y | Y | N/A | N/A | N/A | Y | N/A | Y | Y | Y | Y | Y |
| van den Hoogenband 1984 | Y | N | Y | N/A | N/A | N/A | Y | N/A | Y | Y | Y | N | Y |
| Vaseenon 2012 | N | Y | Y | Y | Y | Y | N/A | N/A | Y | Y | Y | N/A | Y |
| Wenning 2021 | Y | Y | Y | N/A | N/A | N/A | Y | N/A | Y | Y | Y | Y | Y |
| Wiebking 2015 | Y | N | Y | N/A | N/A | N/A | Y | N/A | Y | Y | Y | Y | Y |
| Wilkin 2012 | Y | Y | Y | Y | N/A | Y | Y | N/A | Y | Y | Y | N/A | Y |
| Yokoe 2021 | Y | Y | Y | Y | N/A | N/A | Y | N/A | Y | Y | Y | Y | Y |
| Yokoe 2022 | Y | Y | Y | N/A | Y | N/A | Y | N/A | Y | Y | Y | Y | Y |
| Yokoe 2023 | Y | Y | Y | N/A | Y | N/A | Y | Y | Y | Y | Y | Y | Y |

§: The original text could not be obtained; the data cited from this study were extracted from values reported in the review studies that included the referenced work.

Y: yes; N: no; N/A: not applicable.

Critical appraisal items:

1. If human subjects were used, did the authors give a detailed description of the sample of subjects used to perform the (index) test? 2. Did the authors clarify the qualification, or competence of the rater(s) who performed the (index) test? 3. Was the reference standard explained? 4. If interrater reliability was tested, were raters blinded to the findings of other raters? 5. If intra-rater reliability was tested, were raters blinded to their own prior findings of the test under evaluation? 6. Was the order of examination varied? 7. If human subjects were used, was the time period between the reference standard and the index test short enough to be reasonably sure that the target condition did not change between the two tests? 8. Was the stability (or theoretical stability) of the variable being measured taken into account when determining the suitability of the time interval between repeated measures? 9. Was the reference standard independent of the index test? 10. Was the execution of the (index) test described in sufficient detail to permit replication of the test? 11. Was the execution of the reference standard described in sufficient detail to permit its replication? 12. Were withdrawals from the study explained? 13. Were the statistical methods appropriate for the purpose of the study?

**Limitation:** (1) Stability of variables: Many studies failed to account for the stability of the variables measured during the interval between repeated tests. This oversight may affect the reliability of their findings as participants’ symptoms or function may fluctuate. (2) Order of examination: Several studies did not randomize or vary the order of examinations, which might have introduced order effects or learning effects, potentially impacting the reliability of repeated measurements. (3) Examiner qualifications: The qualifications and expertise of the examiners performing the anterior drawer test were not adequately described in numerous studies, making it difficult to evaluate the competence of individuals conducting the test. (4) Participant withdrawals: Withdrawal mechanisms for participants were insufficiently explained in many cases, which could introduce bias or limit the interpretability of the results. These shortcomings indicate areas for improvement in future research designs to enhance the robustness and reproducibility of findings.

**(F) Characteristics of included studies**

| Study | Participants  (undergoing the drawer test) | Drawer test measurements | | | | | |
| --- | --- | --- | --- | --- | --- | --- | --- |
|  | * Diagnosis &  Demographics | # Examiner qualification | Measuring site | Testing mode | Evaluation method | Reference standard | Reported property |
| ^4^ Ahovuo  1988 | AS: n = 63 (30/33)  26.1 (-), -, - | Unclear | ATFL  CFL | IADT | R: SR  G: SR | Surgery | Accuracy |
| Azni  2020 | Ankle-Injured: n = 42 (20/22)  35.17 (9.45), -, - | Unclear | ATFL | TADT | R: SJ  G: - | AS | Accuracy |
| ^5^ Beumer  2002 | Chronic syndesmotic rupture: n = 3  Control: n = 9 | Expert  Competent | ATFL | TADT | R: SJ  G: - | AS | Reliability  Accuracy |
| ^4^ Blanshard  1986 | n = 142 | Unclear | ATFL  CFL | TADT | R: SJ  G: SR | Surgery  PT | Accuracy  Responsiveness |
| ^4^ Chandnani  1994 | CAI: n = 17 (14/3)  24.5 (20~48), -, - | Competent | ATFL  CFL | TADT | R: SJ  G: SR | MRI  AG | Accuracy  Responsiveness |
| ^2^ Chen  2022 | CAI: n = 153 (83/70)  30.84 (9.43), -, -, BMI: 22.76 (2.34)  Control: n = 160 (73/87)  30.49 (8.11), -, -, BMI: 22.11 (3.01) | Unclear | ATFL | IADT | R: AM  G: AM | US | Reliability  Accuracy  Responsiveness |
| Chen  2023 | MAI: n = 38  31.24 (7.90), 168.93 (7.69), 65.72 (10.47), BMI: 22.91 (2.41)  Control: n = 38  32.10 (7.10), 166.59 (7.89), 62.93 (10.72), BMI: 22.54 (2.40) | Competent | ATFL | IADT | R: AM  G: AM | AS | Accuracy  Responsiveness |
| ^3^ Cho  2016 | CAI: n = 28 (19/9)  32.4 (11.9) | Unclear | ATFL | TADT | R: SJ  G: SR  (150 N) | AS  SR  MRI | Accuracy |
| ^1^ Croy  2013 | Ankle-Injured: n = 66 (35/31)  (ALAS: n = 22, CAI: n = 25, ASC: n = 19)  22.7 (3.6), 174.0 (9.7), 72.5 (14.6) | Expert | ATFL | TADT | R: SJ  G: US  (125 N) | US  AM | Validity  Accuracy  Responsiveness |
| Docherty  2009 | AS: n = 30 (14/16)  20.0 (1.5), 186.7 (10.8), 73.6 (15.9) | Expert  Competent | ATFL | IADT | R: AM  G: AM  (150 N) | - | Reliability  Responsiveness |
| ^3^ Funder  1982 | Ankle-Injured: n = 372 | Unclear | ATFL  CFL | TADT | R: SJ  G: - | AG | Accuracy |
| ^1^ George  2020 | LAS: n = 35 (17/18)  21.97 (7.11), -, - | Competent | ATFL | TADT | R: SJ  G: - | US | Validity  Accuracy |
| ^1^ Gomes  2018 | CAI: n =14 (9/5)  28, -, -  Control: n = 10 | Expert  Competent | ATFL  CFL | TADT | R: SJ  G: - | MRI  SR | Validity  Accuracy |
| ^1^ Großterlinden  2016 | AAS: n = 100 (55/41)  32.6 (10.2) | Expert | ATFL | TADT | R: SJ  G: - | MRI | Reliability  Accuracy |
| Gulick  2024 | AS + Control: n = 30 (10/20)  35.5 (-), -, - | Unclear | ATFL | IADT | R: AM  G: AM | - | Responsiveness |
| ^1^ Hosseinian  2021 | LAS: n = 95 (50/45)  28.5 (8.2), 175.3 (10.1), 72.4 (12.5) | Expert | ATFL | TADT | R: SJ  G: - | MRI | Reliability  Accuracy |
| Iwata  2023 | ATFL-Injured: n = 2  Intact-ATFL: n = 1 | Expert | ATFL | RADT | R: SS  G: US | - | Responsiveness |
| Iwata  2024 | ATFL-Injured: n = 2  Intact-ATFL: n = 2 | Expert  Beginning | ATFL | TADT  RADT | R: SS  G: US | - | Responsiveness |
| ^4^ Johannsen  1978 | Ankle-Injured: n = 244 | Competent | ATFL  CFL | TADT | R: X-ray  G: X-ray | Surgery | Accuracy |
| Kataoka  2022 | n = 4 (3/1)  31.3 (10.1), -, - | Expert | ATFL | TADT | R: EMS  G: EMS | FE | Reliability  Validity  Responsiveness |
| Kawabata  2023 | ALAS: n = 21 (12/9)  17.8 (5.5), -, - | Expert  Competent | ATFL  CFL | RADT | R: US  G: US | - | Reliability  Responsiveness |
| ^1^ Li  2020 | ATFL-Injured: n = 31 (18/13)  30.4 (8.9), -, -, BMI: 22.6 (3.7)  Control: n = 29 (15/14)  29.1 (8.9), -, -, BMI: 22.5 (3.7) | Expert  Competent | ATFL | TADT  ALDT  RALDT | R: SJ  G: - | US | Reliability  Accuracy |
| ^2^ Lin  2013 | AS: n = 8 (6/2)  24.8 (-), -, -  Control: n = 8 (5/3)  24.6 (-), -, - | Unclear | ATFL | IADT | R: SJ  G: AM | - | Reliability |
| ^3^ Lindstrand  1976 | n = 110 | - | - | RALDT | - | Surgery | Accuracy |
| Murahashi  2023 | Ankle-Instability/pain: n = 24 (6/18)  32.0 (23~38), 171.1 (154~185), 65.9 (46~92) | Expert  Competent | ATFL | TADT  IADT | R: SJ  G: AM | SS | Reliability  Accuracy  Responsiveness |
| ^1^ Parasher  2012 | AS + Control: n = 20 (5/15)  20~30, -, - | Unclear | ATFL | TADT | R: AM  G: AM | - | Reliability |
| ^1^ Phisitkul  2009 | Cadaver: n = 10 (6/4)  (Intact ligaments: n = 3, ATFL cut: n = 4, CFL cut: n = 3)  50 (-), -, - | Expert  Competent | ATFL  CFL | TADT  ALDT | R: SJ  G: DAM  (143 N) | DAM | Validity  Accuracy |
| ^3^ Prins  1978 | n = 298 | - | - | TADT | - | AG | Accuracy |
| ^3^ Raatikainen  1992 | AS: n = 188 (144/44)  27 (13~56), -, - | Unclear | ATFL  CFL | TADT | R: SJ  G: - | SR | Accuracy |
| ^4^ Rijke  1990 | Ankle-Injured: n = 26 (21/5) | Unclear | ATFL  CFL | TADT | R: SJ  G: SR | AG  Surgery | Accuracy  Responsiveness |
| Saengsin  2022 | Cadaver: n = 8 (5/3)  66 (59~82) | Unclear | ATFL  CFL | TADT | R: US  G: US  (50/80 N) | FE | Reliability  Validity  Responsiveness |
| Sillevis  2022 | Healthy subjects: n = 47 (24/23)  30.36 (22~54), -, - | Expert | ATFL  CFL | TADT | R: US  G: US | US | Validity  Responsiveness |
| Song  2021 | GJH: n = 20 (3/17)  28.3 (20~40), 164.0 (158.0~177.0), 64.0 (48.0~88.0), BMI: 23.2 (19.0~30.8)  Control: n = 24 (6/18)  26.9 (20~40), 165.7 (155.0~188.0), 62.4 (47.0~100.0), BMI: 22.9 (17.2~33.2) | Expert | ATFL | TADT | R: SJ  G: - | US | Reliability  Validity  Responsiveness |
| ^2^ Spahn  2004 | ALAS: n = 45 (25/20)  28.8 (10.1), -, - | Unclear | AJ | IADT | R: AM  G: AM | SR | Validity  Responsiveness |
| Teramoto  2021 | Cadaver: n = 5 (0/5)  87.6 (83~93), -, -  Ankle-Instability/pain: n = 20 (15/5)  31.3 (22~55), 170.0 (154~188), 65.1 (46~89.5), BMI: 22.4 (17.1~31.7) | Expert | ATFL  CFL | TADT | R: sensor  G: sensor | SR | Reliability  Validity  Responsiveness |
| ^1^ van Dijk  1996 | ALI: n = 160 (116/44)  27.3 (-), -, - | Expert  Competent | ATFL | TADT | R: SJ  G: - | AG  SF | Accuracy |
| ^2^ van Dijk  1996 (b) | ALI: n = 160 (116/44)  18~40, -, - | Expert  Competent | ATFL | TADT | R: SJ  G: - | AG  SF | Accuracy |
| ^3^ van den Hoogenband  1984 | AS: n = 150 | Unclear | ATFL  CFL | TADT | R: SJ  G: - | Surgery | Accuracy |
| ^1^ Vaseenon  2012 | Cadaver: n = 9 (2/3)  55 (48~70), -, - | Expert  Competent | ATFL  CFL | TADT  ALDT | R: SJ  G: DAM | DAM | Reliability  Validity  Accuracy  Responsiveness |
| ^2^ Wenning  2021 | MAI: n = 25  24.6 (4.7), -, -, BMI: 23.0 (3.5)  Control: n = 25  23.6 (4.0), -, -, BMI: 21.9 (2.4) | Expert | ATFL  CFL | TADT | R: SJ  G: AM  (150 N) | US  MRI | Validity |
| ^2^ Wiebking  2015 | AS: n = 30 (17/13)  35 (14), -, - | Unclear | ATFL | TADT  IADT | R: SJ  G: AM  (100 N) | US  SS | Accuracy  Responsiveness |
| ^1^ Wilkin  2012 | n = 60 (9/51)  (AS: n = 38, non-AS: 22)  17~50, -, - | Expert  Beginning | ATFL | TADT  RADT | R: SJ  G: - | - | Reliability  Validity |
| Yokoe  2021 | Ankle non-injured: n = 184 (96/88)  24.5 (2.7), 165.4 (9.4), 59.6 (11.2), BMI: 21.6 (2.6) | Expert | ATFL | TADT | R: SJ  G: US | US | Validity  Responsiveness |
| Yokoe  2022 | Beighton score ≥ 6: n = 20 (0/20)  24.3 (1.8), 159.5 (5.2), 52.4 (4.8), BMI: 20.8 (1.5)  Beighton score ≤ 3: n = 61 (0/61)  23.7 (2.1), 157.5 (5.7), 50.3 (4.6), BMI: 20.3 (1.4) | Expert | ATFL | TADT | R: SJ  G: - | - | Reliability  Validity  Responsiveness |
| Yokoe  2023 | GJL: n = 13 (13/0)  25.5 (2.6), 171.0 (4.2), 64.0 (10.6), BMI: 22.2 (2.8)  No GJL: n = 95 (95/0)  25.0 (2.9), 173.0 (5.2), 66.4 (6.7), BMI: 22.8 (3.0) | Expert | ATFL | TADT | R: SJ  G: - | - | Validity  Responsiveness |

^1, 2, 3, 4, 5^: Some of the data cited from this reference in the current study are derived from the values calculated and reported in the review that included this reference (Review 1: Beynon 2022, Review 2: Schurz 2023, Review 3: Netterström-Wedin 2021, Review 4: Frost 1999, Review 5: Sman 2013).

*: The group names reported in the diagnosis primarily pertain to injury condition/type, including: AS: ankle sprain, ALI: ankle ligament injury, AAS: acute ankle sprain, LAS: lateral ankle sprain, LALI: lateral ankle ligament injury, ALAS: acute lateral ankle sprain, ASC: ankle sprain coper, CAI: chronic ankle instability, MAI: mechanical ankle instability, GJH: generalized joint hypermobility, GJL: generalized joint laxity. The content reported under demographics includes: number (n), gender (male/female), age (year, mean (SD)/(range)), height (cm, mean (SD)/(range)), Weight (kg, mean (SD)/(range)), BMI (mean (SD)/(range)).

#: Expert: highly experienced senior expert; Competent: some experience, has a basic understanding, not a complete novice but not yet highly proficient; Beginning: complete novice, beginner.

ATFL: anterior talofbular ligament, CFL: calcaneofibular ligament, AJ: ankle joint, ADT: anterior drawer test, TADT: traditional anterior drawer test, ALDT: anterolateral drawer test, RADT: reverse anterior drawer test, RALDT: reverse anterolateral drawer test, IADT: instrumented anterior drawer test, TTT: talar tilt test, R: rating (for grades), G: gauge (for displacements), US: ultrasound imaging, AM: arthrometer (auto/ manual), GM: goniometer (ruler/ankle meter), DAM: direct anatomical measurement, AG: arthrography, AS: arthroscopy, SJ: subjective judgment, ME: mechanical testing, SR: stress radiography, SS: stress sonography, MRI: magnetic resonance imaging, SF: surgical findings, PT: peroneal tenography, EMS: electromagnetic measurement system, FE: fluoroscopic evaluation.
