# Supplementary material for: Is the anterior drawer test still valuable for diagnosing mechanical ankle instability in clinical practice and research?
Source: Front Bioeng Biotechnol. 2025 Sep 9;13:1664779. doi: 10.3389/fbioe.2025.1664779 (PMC12454323; doi:10.3389/fbioe.2025.1664779)
Supplement: Supplementary file 2 [file Table2.docx]

**(A) Summary of Reliability Reporting in Included Studies**

| Study | Test-Retest Reliability (Intra-rater reliability) | Inter-Rater Reliability |
| --- | --- | --- |
| ***Anterior drawer test*** | | |
| ^5^ Beumer  2002 |  | ICC 0.059 (-0.19, 0.205) |
| ^1^ Großterlinden  2016 |  | Kappa 0.366 |
| ^1^ Hosseinian  2021 |  | Kappa 0.356 (Sprain + Partial tear + Complete tear)  Kappa 0.461 (Partial tear + Complete tear)  Kappa 0.349 (Complete tear) |
| Murahashi  2023 |  | Kappa 0.416 (0.096-0.735) |
| Kataoka  2022 | ICC 0.99 | ICC 0.89 |
| ^1^ Li  2020 |  | Kappa 0.196 |
| ^1^ Parasher  2012 | Tester 1: ICC 0.96 (0.94, 0.98), SEM: 0.72, LOA: 0.19  Tester 2: ICC 0.97 (0.96, 0.98), SEM: 0.6, LOA: 0.15 | ICC 0.70 (0.48, 0.82), SEM: 2.27, LOA: 0.54 |
| Saengsin  2022 | Anterior drawer test + talar tilt test  ICC 0.97 (0.95-0.99)  (portable ultrasonography)  ICC 0.99 (0.99-1.00)  (fluoroscopy) | Anterior drawer test + talar tilt test  ICC 0.99 (0.98-0.99)  (portable ultrasonography)  ICC 0.99 (0.99-1.00)  (fluoroscopy) |
| Song  2021 | ICC > 0.75 | ICC > 0.75 |
| Teramoto  2021 | Cadavers:  ICC 0.865 (investigator 1), ICC 0.939 (investigator 2), ICC 0.862 (investigator 3)  Patients: ICC 0.906 | Cadavers: ICC 0.815 (3 investigators)  Patients: ICC 0.95 |
| ^1^ Vaseenon  2012 | ICC 0.9443 | ICC 0.5274 |
| ^1^ Wilkin  2012 | The 80% percent close agreement among the three raters for the anterior drawer test in supine | Between the 3 raters (experienced and student): ICC 0.16 (0.10, 0.33), SEM: 1.11  Experienced raters: ICC 0.23 (-0.02, 0.46), SEM: 1.19 |
| Yokoe  2022 | Kappa 0.83 |  |
| ***Anterolateral drawer test*** | | |
| ^1^ Li  2020 |  | Kappa 0.528 |
| ^1^ Vaseenon  2012 | ICC 0.8017 | ICC 0.5230 |
| ***Reverse anterior drawer test*** | | |
| Kawabata  2023 |  | Manual tester A vs. B  ICC 0.81 (0.68-0.89) minimal distance  ICC 0.80 (0.64-0.89) maximal distance  ICC 0.67 (0.43-0.81) maximal-minimal distance  Automated Length Measurement System (ALMS) vs. tester A  ICC 0.63 (0.41-0.79) minimal distance  ICC 0.63 (0.41-0.78) maximal distance  ICC 0.53 (0.07-0.77) maximal-minimal distance  Automated Length Measurement System (ALMS) vs. tester B  ICC 0.62 (0.38-0.77) minimal distance  ICC 0.71 (0.56-0.83) maximal distance  ICC 0.59 (0.32-0.77) maximal-minimal distance |
| ^1^ Li  2020 |  | Kappa 0.639 |
| ^1^ Wilkin  2012 | The absolute agreement among the three raters ranged from 5% for the anterior drawer in crook lying | Between the 3 raters (experienced and student): ICC 0.06 (−0.08, 0.23), SEM: 1.39  Experienced raters: ICC -0.12 (-0.36, 0.14), SEM: 1.69 |
| ***Instrumented anterior drawer test*** | | |
| ^2^ Chen  2022 | ICC 0.897 (95% CI, 0.227–0.969) (a single measure)  ICC 0.963 (95% CI, 0.469–0.989) (the average of 3 measures) | ICC 0.949 (2 independent examiners) |
| Docherty  2009 | ICC 0.65, SEM 2.22 | ICC 0.81, SEM 1.61 |
| Murahashi  2023 |  | Kappa 0.571 (0.265-0.878) |
| ^2^ Lin 2013 | ICC > 0.90  (for any two loops with the same maximum force) |  |

^1, 2, 3, 4, 5^: Some of the data cited from this reference in the current study are derived from the values calculated and reported in the review that included this reference (Review 1: Beynon 2022, Review 2: Schurz 2023, Review 3: Netterström-Wedin 2021, Review 4: Frost 1999, Review 5: Sman 2013).

ICC: intraclass correlation coefficient, Kappa: Cohen's Kappa coefficient, SEM: standard error of measurement, LOA: Limits of Agreement.

**(B) Summary of Validity Reporting in Included Studies**

| Study | Construct Validity  (Convergent/Discriminant) | Criterion Validity  (Concurrent/Predictive) |
| --- | --- | --- |
| ***Anterior drawer test*** | | |
| ^1^ Croy  2013 |  | There was a moderately strong correlation between the clinical grades of laxity given with the manually performed ADT and the amount of anterior laxity measured from the US images for the group with an acute ankle sprain (rho = 0.62, *p* = 0.002). No significant relationship was observed between these 2 variables for the copers (rho = -0.11, *p* = 0.67) or those with CAI (rho = 0.10, *p* = 0.62). |
| ^1^ George  2020 |  | Clinical vs. ultrasound grading  spearman’s ρ, *p* value, significance and strength of correlation:  ATFL (overall), 0.58, <0.001, significant, moderate  ATFL (in male), 0.51, 0.04, significant, moderate  ATFL (in female), 0.66, 0.03, significant, moderate  ATFL (in adolescent), 0.90, <0.001, significant, very strong  ATFL (in adult), 0.31, 0.20, non-significant |
| ^1^ Gomes  2018 | Anterolateral talar palpation test  Kappa 0.32; *p* = 0.036 | Agreement with MRI magnetic resonance imaging  MRI, Kappa 0.44; *p* = 0.011  X-ray, Kappa 0.57; *p* = 0.002 |
| Kataoka  2022 |  | There was a significant positive correlation between the anterior translation of the ankle joint measured using EMS and radiography (fluoroscope) (*p* < 0.01). The correlation coefficient was 0.91. |
| ^1^ Phisitkul  2009 |  | DAM, r = 0.519, *p* = 0.117 |
| Saengsin  2022 |  | When evaluated the correlations between portable‐US and fluoroscopic measurements, strong positive correlations between portable‐US and fluoroscopic values measured during 50N and 80N of ADT were found (r = 0.85, 95% CI: 0.68–0.93, *p* < 0.001 and r = 0.81, 95% CI: 0.60–0.92, *p* < 0.001, respectively). |
| Sillevis  2022 | Age, ATFL: r = -0.130, *p* = 0.38  Age, CFL: r -0.394, *p* = 0.006 |  |
| Song  2021 | Beighton score, r = 0.719, *p* < 0.01  ATFL resting  length / height / thickness:  r = 0.638, *p* <0.01  r = 0.763, *p* <0.01  r = 0.232  ATFL stress  length / height / thickness:  r = 0.750, *p* <0.01  r = 0.696, *p* <0.01  r = 0.419, *p* <0.01 |  |
| Teramoto  2021 |  | The correlation coefficient between the sensor and the radiographic images was 0.843 (95% CI, 0.65- 0.93; *p* < 0.001) |
| ^1^ Vaseenon  2012 |  | ADT and DAM (r = 0.7621, strong) in orthopaedic trainees  ADT and DAM (r = 0.0208, weak) in athletic training students  ADT and DAM (r = 0.5704, strong) in both |
| ^2^ Wenning  2021 | ADT & CAIT:  rho -0.81 (-0.63, -0.88)  ADT & FJS:  rho -0.75 (-0.57, -0.87)  (ADT+SS) & CAIT:  rho -0.48 (-0.2, -0.72)  (ADT+SS) & FJS:  rho -0.35 (-0.02, -0.65) | Cartilage contact area (CCA) in the fibulotalar joint (CCAFT: cartilage contact area fibulotalar) as well as the horizontal (CCATTH: cartilage contact area tibiotalar horizontal) and vertical (CCATTV: cartilage contact area tibiotalar vertical) parts of the CCA in the tibiotalar joint were measured.  ADT & 3D stress ankle MRI (CCAFT/CCATTH/CCATTV):  rho 0.48 (0.26, 0.7), 0.34 (-0.1, 0.45), n.s.  (ADT+SS) & 3D stress ankle MRI (CCAFT/CCATTH/CCATTV):  rho -0.30 (-0.05, 0.44), n.s., n.s.  ADT & (ADT+SS):  rho 0.47 (0.12, 0.65) |
| ^1^ Wilkin  2012 | ADT in supine & CAIT:  Novice rater: r = 0.016  Experienced rater: r = -0.124 |  |
| Yokoe  2021 |  | When comparing patients with grade 1 versus grade 2 ADT, there was a significant difference in the ATFL ratio for all ankles (1.10 ± 0.06 vs 1.08 ± 0.03, *p* < 0.001) and for male ankles (1.11 ± 0.06 vs 1.07 ± 0.03, *p* = 0.002); there was no significant difference in female ankles (1.10 ± 0.05 vs 1.08 ± 0.04, *p* = 0.12).  Patients with GJL showed a significantly higher rate of grade 2 ADT than those without GJL. When comparing patients with versus without GJL, there was a significant difference in the ATFL ratio for all ankles (1.10 ± 0.05 vs 1.08 ± 0.04; *p* = 0.003) and for male ankles (1.11 ± 0.06 vs 1.07 ± 0.03, *p* = 0.02). There was no significant difference in female ankles (1.10 ± 0.05 vs 1.09 ± 0.04, *p* = 0.24). |
| Yokoe  2022 | A correlation between the Beighton score and nonstress ATFL length was not detected (r = 0.01, *p* = 0.90), nor was any correlation detected between the BS and stress ATFL length (r = 0.07, *p* = 0.52) or between the BS and ATFL ratio (r = 0.15, *p* = 0.19). |  |
| Yokoe  2023 | A weak correlation between the BS and nonstress ATFL length was detected (r = 0.21, *p* = 0.03). Moderate correlations were identified between the BS and stress ATFL length (r = 0.43, *p* < 0.0001) and between the BS and ATFL ratio (r = 0.45, *p* < 0.0001). |  |
| ***Anterolateral drawer test*** | | |
| ^1^ Phisitkul  2009 |  | ALDT & DAM:  r = 0.931 *p* = 0.001 |
| ^1^ Vaseenon  2012 |  | ALDT & DAM in orthopaedic trainees:  r = 0.7656, strong  ALDT & DAM in athletic training students:  r = 0.6129, strong  ALDT & DAM in both:  r = 0.7332, strong |
| ^1^ Wilkin  2012 | ADT in crook lying & CAIT:  Novice rater: r = 0.088  Experienced rater: r = -0.141 |  |
| ***Instrumented*** ***anterior drawer test*** | | |
| ^2^ Spahn  2004 | The VAS-pain did not correlate with the results of ADT in clinical (r = 0.12) or radiological (r = 0.15) tests. | An ADT of 8.9±4.3 mm was measured in the ankles after sprain in clinical test. In stress radiography ADT was 9.9±4.9 mm. The results of these values correlated significantly (r = 0.911, *p* < 0.05). |

^1, 2, 3, 4, 5^: Some of the data cited from this reference in the current study are derived from the values calculated and reported in the review that included this reference (Review 1: Beynon 2022, Review 2: Schurz 2023, Review 3: Netterström-Wedin 2021, Review 4: Frost 1999, Review 5: Sman 2013).

Construct validity includes convergent validity and divergent validity. Criterion validity primarily involves comparisons between the drawer test and imaging or anatomical observations. The measurement units used are: Pearson Correlation Coefficient, represented by “r”, and Spearman’s Rank Correlation Coefficient, represented by “ρ/rho”.

ADT: ankle drawer test, US: ultrasound, CAI: chronic ankle instability, ATFL: anterior talofbular ligament, MRI: magnetic resonance imaging, EMS: electromagnetic measurement system, DAM: direct anatomical measurement, BS: Beighton score, GJL: generalized joint laxity, ALDT: anterolateral drawer test, ATD: anterior talar drawer, FJS: forgotten Joint Score, CAIT: Cumberland ankle instability tool, SS: stress sonography, VAS: visual analogue scale.

**(C) Summary of Accuracy Reporting in Included Studies**

| Study | Sensitivity  (cut-off) | Specificity  (cut-off) |
| --- | --- | --- |
| ***Anterior drawer test*** | | |
| Azni  2020 | 77.8 | 0 |
| ^5^ Beumer  2002 | 36 (16, 61) | 43 (16, 75) |
| ^4^ Blanshard  1986 | Stress views versus surgery: 32  Stress views versus tenography: 32 | Stress views versus surgery: 100  Stress views versus tenography: 100 |
| ^4^ Chandnani  1994 | Tear: 57 | Tear: 100 |
| ^3^ Cho  2016 | 79 (59, 92) | Not estimable |
| ^1^ Croy  2013 | Grade 2 or above considered positive (2.3 mm or greater):  74 (58, 86)  Grade 2 or above considered positive (3.7 mm or greater):  83 (64, 93)  Grade 3 or above considered positive (2.3 mm or greater):  26 (14, 42)  Grade 3 or above considered positive (3.7 mm or greater):  33 (18, 53) | Grade 2 or above considered positive (2.3 mm or greater):  38 (24, 56)  Grade 2 or above considered positive (3.7 mm or greater):  40 (27, 56)  Grade 3 or above considered positive (2.3 mm or greater):  67 (50, 81)  Grade 3 or above considered positive (3.7 mm or greater):  73 (59, 85) |
| ^3^ Funder  1982 | 12 (8, 17) | 94 (89, 97) |
| ^1^ George  2020 | Ultrasound grade 3 tear was taken as reference standard:  59 (36, 79) | Ultrasound grade 3 tear was taken as reference standard:  100 (75, 100) |
| ^1^ Gomes  2018 | 50 | 100 |
| ^1^ Großterlinden  2016 | 44.4 | 67.8 |
| ^1^ Hosseinian  2021 | Sprain + Partial tear + Complete tear: 81; 100 (+ US)  Partial tear + Complete tear: 85; 96 (+ US)  Complete tear: 42 | Sprain + Partial tear + Complete tear: 80; 80 (+ US)  Partial tear + Complete tear: 63; 50 (+ US)  Complete tear: 94 |
| Murahashi  2023 | Junior examiner: 40.0 (12.2, 73.8)  Senior examiner: 80.0 (44.4, 97.5) | Junior examiner: 75.0 (50.9, 91.3)  Senior examiner: 60.0 (36.1, 80.9) |
| ^4^ Johannsen  1978 | 74 | 0 |
| ^1^ Li  2020 | Junior examiner: 5.3  Senior examiner: 39.5  Overall: 22 (14, 33) | Junior examiner: 100  Senior examiner: 100  Overall: 100 (95, 100) |
| ^1^ Phisitkul  2009 | 75 | 50 |
| ^3^ Prins  1978 | 45 (38, 52) | 78 (69, 86) |
| ^3^ Raatikainen  1992 | Arthrography (reference standard): 75 (66, 82)  Surgery (reference standard): 53 | Arthrography (reference standard): 67 (53, 79)  Surgery (reference standard): Not estimable |
| ^4^ Rijke  1990 | Excluding nine patients with history of ankle injury on opposite ankle: 32  Opposite ankle: 31 | Excluding nine patients with history of ankle injury on opposite ankle: 91  Opposite ankle: 100 |
| ^3^ van den Hoogenband  1984 | 79 (71, 85) | Not estimable |
| ^1^ van Dijk  1996 | Physical examination within 48 hours of injury: 71  Physical examination five days after injury: 96 | Physical examination within 48 hours of injury: 33  Physical examination five days after injury: 84 |
| ^2^ van Dijk  1996 (b) | Physical examination by an emergency physician within 48 hours after trauma: 71  Delayed physical examination performed by an experienced orthopedic surgeon: 96  Delayed physical examination performed by 1 of 4 inexperienced but well-instructed physicians: 89 | Physical examination by an emergency physician within 48 hours after trauma: 33  Delayed physical examination performed by an experienced orthopedic surgeon: 84  Delayed physical examination performed by 1 of 4 inexperienced but well-instructed physicians: 70 |
| ^1^ Vaseenon  2012 | 100 | 66.67 |
| ^2^ Wiebking  2015 | 93 | 67 |
| ***Anterolateral drawer test*** | | |
| ^1^ Li  2020 | Junior examiner: 44.7  Senior examiner: 50  Overall: 47 (36, 59) | Junior examiner: 100  Senior examiner: 97.1  Overall: 99 (92, 100) |
| ^1^ Phisitkul  2009 | 100 | 100 |
| ^1^ Vaseenon  2012 | 100 | 66.67 |
| ***Reverse anterior drawer test*** | | |
| ^1^ Li  2020 | Junior examiner: 86.8  Senior examiner: 92.1  Overall: 89 (80, 95) | Junior examiner: 91.2  Senior examiner: 88.2  Overall: 90 (80, 96) |
| ^3^ Lindstrand  1976 | 83 (73, 90) | 70 (47, 87) |
| ***Instrumented anterior drawer test*** | | |
| ^4^ Ahovuo  1988 | 54 | 88 |
| ^2^ Chen  2022 | 87.3 (80.4, 92.0)  (Displacement at 75N, cutoff 8.15mm)  80.4 (73.4, 85.9)  (Load-displacement ratio at interval of 10-40N, cutoff 0.1582 mm/N) | 71.9 (63.7, 78.8)  (Displacement at 75N, cutoff 8.15mm)  86.3 (80.1, 90.7)  (Load-displacement ratio at interval of 10-40N, cutoff 0.1582 mm/N) |
| Chen  2023 | 80 (62.7, 90.5)  (Displacement at 75N, cutoff 1.85mm)  90.0 (74.4, 96.5)  (Load-displacement ratio at interval of 10-40N, cutoff 0.0351 mm/N) | 93.3 (78.7, 98.8)  (Displacement at 75N, cutoff 1.85mm)  93.3 (78.7, 98.8)  (Load-displacement ratio at interval of 10-40N, cutoff 0.0351 mm/N) |
| Murahashi  2023 | Junior examiner: 80.0 (44.4, 97.5)  Senior examiner: 70.0 (34.8, 93.3) | Junior examiner: 80.0 (56.3, 94.3)  Senior examiner: 85.0 (62.1, 96.8) |
| ^2^ Wiebking  2015 | 80 (Using a 3.9 mm cut-off) | 40 (Using a 3.9 mm cut-off) |

^1, 2, 3, 4, 5^: Some of the data cited from this reference in the current study are derived from the values calculated and reported in the review that included this reference (Review 1: Beynon 2022, Review 2: Schurz 2023, Review 3: Netterström-Wedin 2021, Review 4: Frost 1999, Review 5: Sman 2013).

US: ultrasound imaging, AM: arthrometer (auto/ manual), GM: goniometer (ruler/ankle meter), DAM: direct anatomical measurement, AG: arthrography, AS: arthroscopy, SJ: subjective judgment, ME: mechanical testing, SR: stress radiography, SS: stress sonography, MRI: magnetic resonance imaging, SF: surgical findings, PT: peroneal tenography, EMS: electromagnetic measurement system, FE: fluoroscopic evaluation.

**(D) Summary of Responsiveness Reporting in Included Studies**

| Study | Displacement gauge (cut-off) |
| --- | --- |
| ***Anterior drawer test*** | |
| ^4^ Blanshard  1986 | Normal ankles (n = 216), 0.8 (0~4) mm  Abnormal tenograms (n = 65), 2.6 (0~9) mm  Normal tenograms (n = 77), 1.9 (0~7) mm |
| ^4^ Chandnani  1994 | Tibiotalar length of separation measured after application of anterior drawer stress were 3~12 mm. |
| ^1^ Croy  2013 | The mean ± SD anterior laxity measured when applying 125 N of force with the arthrometer was 3.36 ± 3.25 mm for the ankle-injured group (n = 66) and 0.17 ± 1.87 mm for the controls (n = 20). Only 1 subject (5%) in the control group had laxity greater than both specified thresholds (2.3 and 3.7 mm). |
| Murahashi  2023 | The displacement distance of the ADT measured by the junior examiner is significantly lower than that of the senior examiner (3.26 ± 2.03 mm vs. 4.5 ± 1.88 mm, *p* = 0.017) |
| Iwata  2024 | Patient 1 (intact ATFL):  Experienced: 0.6 (0.1~1.0) mm; Beginner: 0.4 (-0.5~2.0) mm  Patient 2 (injured ATFL):  Experienced: 3.2 (0.9~5.0) mm; Beginner: 3.7 (1.9~6.0) mm  In the ROC curve analysis, the optimal cut-off value was 1.7 mm, with the area under the curve of 0.98. |
| Kataoka  2022 | ADT by EMS was 8.1 ± 5.7 mm, range: 2.6~21.0 mm; by radiographic evaluation was 3.6 ± 2.4 mm, range: 1.4~9.0 mm.  The anterior translation of the three examiners averaged 5.0 ± 2.2 mm, 5.7 ± 2.7 mm, and 5.2 ± 2.7 mm.  The mean anterior translation for the foot with and without a history of sprain was 6.4 ± 2.2 mm and 2.7 ± 1.8 mm, indicating a significant difference (*p* < 0.001). |
| ^4^ Rijke  1990 | Anterior talar displacement: Injured: 5.2 (3.0), range: 2~12 mm; Opposite: 3.4 (1.7), range: 1~8 mm  Opposite excluding the 9 patients with a positive history on the opposite ankle: 2.8 (0.9), range: 1~4 mm |
| Saengsin  2022 | Sequence of lateral ligament transection Portable‐US anterior talar translation at 50‐N in mm (median, IQR) Portable‐US anterior talar translation at 80‐N in mm (median, IQR):  Intact: 0.88 (0.4, 1.4), 1.3 (0.7, 1.6); ATFL: 2.8 (2.3, 4.2), 3.9 (3.0, 5.5); ATFL, CFL: 4.6 (3.8, 4.9), 5.5 (4.9, 6.5);  ATFL, CFL, PTFL: 6.0 (3.6, 7.1), 7.8 (5.0, 9.2).  The sequence of lateral ligament transection Fluoroscopic anterior talar translation at 50‐N in mm (median, IQR) Fluoroscopic anterior talar translation at 80‐N in mm (median, IQR):  Intact: 2.5 (1.8, 3.5), 4.1 (3.1, 5.2); ATFL: 4.5 (2.8, 5.7), 7.6 (4.7, 8.5); ATFL, CFL: 6.4 (5.5, 6.8), 8.4 (7.9, 10.1);  ATFL, CFL, PTFL: 7.4 (6.0, 9.2), 10.1 (8.9, 10.8). |
| Sillevis  2022 | ATFL: 2.7332 cm (relaxed 2.5562 cm)  CFL: 2.5211 cm (relaxed 2.4485 cm) |
| Song  2021 | GJH:  ATFL length difference between resting and stress: 0.7 (0.3~1.8) mm  ATFL height difference between resting and stress: 0.9 (0.4~1.3) mm  ATFL thickness difference between resting and stress: 0.2 (0.0~0.5) mm  Control:  ATFL length difference between resting and stress: 1.2 (0.0~3.1) mm  ATFL height difference between resting and stress: 0.1 (0.0~0.7) mm  ATFL thickness difference between resting and stress: 0.1 (0.0~0.4) mm |
| Teramoto  2021 | Cadavers:  The mean anterior drawer distance for the intact ankles was 3.7 ± 1.0 mm.  This increased significantly to 6.1 ± 1.6 mm after the ATFL was transected (*p* < 0.001). When both the ATFL and CFL had been transected, the value was 7.9 ± 1.8 mm, which was a significant increase compared with the intact ankles (*p* < 0.001) and ankles with transection of the ATFL alone (*p* = 0.006).  Patients:  The mean anterior drawer distance measured by the sensor was 2.9 ± 0.9 mm.  The mean anterior drawer displacement measured from the radiographic images was 2.7 ± 0.9 mm. |
| ^1^ Vaseenon  2012 | Orthopaedic trainees (Intact, ATFL cut, ATFL+CFL cut): 3.2 ± 1.4, 6.5 ± 1.5, 6.8 ± 1.2  Athletic training students (Intact, ATFL cut, ATFL+CFL cut): 4.5 ± 1.6, 4.3 ± 1.1, 3.8 ± 1.6  Both (Intact, ATFL cut, ATFL+CFL cut): 3.9 ± 1.6, 5.4 ± 1.7, 5.3 ± 2.1 |
| Yokoe  2021 | All (ATFL nonstress length, ATFL stress length, ATFL ratio (ATFL stress / ​ATFL nonstress)​​):  19.50 ± 1.81, 21.08 ± 1.98, 1.08 ± 0.04  Male (ATFL nonstress length, ATFL stress length, ATFL ratio (ATFL stress / ​ATFL nonstress)​​):  20.41 ± 1.62, 21.92 ± 1.86, 1.07 ± 0.04  Female (ATFL nonstress length, ATFL stress length, ATFL ratio (ATFL stress / ​ATFL nonstress)​​):  18.57 ± 1.49, 20.22 ± 1.70, 1.09 ± 0.04 |
| Yokoe  2022 | Low Beighton score (ATFL nonstress length, ATFL stress length, ATFL ratio (ATFL stress / ​ATFL nonstress)​​):  18.2 ± 1.5, 19.9 ± 1.7, 1.09 ± 0.05  High Beighton score (ATFL nonstress length, ATFL stress length, ATFL ratio (ATFL stress / ​ATFL nonstress)​​):  18.1 ± 1.1, 20.0 ± 1.4, 1.10 ± 0.05 |
| Yokoe  2022 | No generalized joint laxity (ATFL nonstress length, ATFL stress length, ATFL ratio (ATFL stress / ​ATFL nonstress)​​):  20.4 ± 1.6, 21.7 ± 1.8, 1.07 ± 0.03  Generalized joint laxity (ATFL nonstress length, ATFL stress length, ATFL ratio (ATFL stress / ​ATFL nonstress)​​):  20.5 ± 1.5, 23.6 ± 1.8, 1.15 ± 0.06 |
| ***Anterolateral drawer test*** | |
| ^1^ Vaseenon  2012 | Orthopaedic trainees (Intact, ATFL cut, ATFL+CFL cut): 2.8 ± 0.5, 5.0 ± 1.8, 6.1 ± 1.5  Athletic training students (Intact, ATFL cut, ATFL+CFL cut): 4.0 ± 0.4, 5.5 ± 1.7, 5.4 ± 2.6  Both (Intact, ATFL cut, ATFL+CFL cut): 3.4 ± 0.7, 5.2 ± 1.7, 5.8 ± 2.0 |
| ***Reverse anterior drawer test*** | |
| Iwata  2023 | Patient 1 (intact ATFL):  Preferred technique: 0.4 (-2.3~1.3) mm; Standardized technique: 0.6 (-0.6~1.7) mm  Patient 2 (injured ATFL):  Preferred technique: 2.0 (0.3~4.4) mm; Standardized technique: 1.7 (-0.9~3.8) mm  Patient 3 (injured ATFL):  Preferred technique: 1.4 (-2.7~7.1) mm; Standardized technique: 0.7 (-2.0~2.3) mm |
| Iwata  2024 | Patient 1 (intact ATFL):  Experienced: 0.5 (0.1~0.9) mm; Beginner: 0.4 (-0.1~1.5) mm  Patient 2 (injured ATFL):  Experienced: 3.2 (2.0~5.0) mm; Beginner: 2.3 (0.0~4.1) mm  In the ROC curve analysis, the optimal cut-off value was 2.0 mm, with the area under the curve of 0.95. |
| Kawabata  2023 | Manual tester A:  Unaffected side (Minimal distance, Maximal distance, Max-min): 21.34 (3.14) mm, 23.14 (3.23) mm, 1.80 (0.92) mm  Affected side (Minimal distance, Maximal distance, Max-min): 22.60 (2.20) mm, 25.87 (2.82) mm, 3.27 (1.39) mm  Manual tester B:  Unaffected side (Minimal distance, Maximal distance, Max-min): 21.29 (2.96) mm, 22.72 (2.97) mm, 1.43 (0.85) mm  Affected side (Minimal distance, Maximal distance, Max-min): 22.27 (2.34) mm, 25.03 (2.53) mm, 2.76 (1.01) mm  Automated Length Measurement System (ALMS) in ultrasonography:  Unaffected side (Minimal distance, Maximal distance, Max-min): 22.59 (3.48) mm, 23.48 (3.58) mm, 0.89 (0.60) mm  Affected side (Minimal distance, Maximal distance, Max-min): 22.84 (2.61) mm, 25.15 (2.91) mm, 2.30 (1.43) mm |
| ***Instrumented anterior drawer test*** | |
| ^2^ Chen  2022 | Displacement at 75N, cutoff: 8.15 mm  Load-displacement ratio at interval of 10-40N, cutoff: 0.1582 mm/N |
| Chen  2023 | Displacement at 75N, cutoff: 1.85 mm  Load-displacement ratio at interval of 10-40N, cutoff: 0.0351 mm/N |
| Docherty  2009 | Investigator 1 on day 1: 20.01 (3.72) mm  Investigator 1 on day 2: 19.21 (2.38) mm  Investigator 2: 18.71 (2.57) mm |
| Gulick  2024 | Differences between injured (sprained) & uninjured ankles (Control, Grade 1, Grade 2):  0.31 ± 0.47 mm, 1.11 ± 0.52 mm, 2.16 ± 0.85 mm  Normal translation of ADT has been reported to be from 3~10 mm with a mean of 2.00 mm ± 1.71 mm using stress radiographs. |
| Murahashi  2023 | There is no significant difference in the displacement distance of the ADT between junior and senior examiners:  4.64 ± 2.08 mm vs. 4.14 ± 1.96 mm, *p* = 0.340 |
| ^2^ Spahn  2004 | The ATD of all examined ankles (n = 76) was 1.7± 1.3 mm. A total of 38 healthy volunteers were available as control group. The ATD in the control group was 1.6±1.7 mm in right and 1.7±1.2 mm in left ankles (no significance). The ATD than in female subjects (right side 2.3±1.2 mm, left side 2.2±1.1 mm) was significantly higher than in male subjects (right side 0.9±0.8 mm, left side 0.8±0.8 mm). In patients who had suffered from an ankle sprain the ATD of the non-injured ankle was 1.6±1.2 mm. There was no significance in comparison with the control group. There was measured an ATD of 8.9±4.3 mm in the ankles after sprain. This difference was significant. In the control group no difference was detected between the right and left ankles. The difference between healthy and injured ankle in case of an ankle sprain was 7.4±4.2 mm. |
| ^2^ Wiebking  2015 | Cut-off: 3.9 mm |

^1, 2, 3, 4, 5^: Some of the data cited from this reference in the current study are derived from the values calculated and reported in the review that included this reference (Review 1: Beynon 2022, Review 2: Schurz 2023, Review 3: Netterström-Wedin 2021, Review 4: Frost 1999, Review 5: Sman 2013).

ADT: anterior drawer test, ATFL: anterior talofbular ligament, EMS: electromagnetic measurement system, US: ultrasound imaging, IQR: interquartile ranges, CFL: calcaneofibular ligament, PTFL: posterior talofibular ligament, GJH: generalized joint hypermobility, GJL: generalized joint laxity, AM: arthrometer (auto/ manual), GM: goniometer (ruler/ankle meter), DAM: direct anatomical measurement, AG: arthrography, AS: arthroscopy, SJ: subjective judgment, ME: mechanical testing, SR: stress radiography, SS: stress sonography, MRI: magnetic resonance imaging, SF: surgical findings, PT: peroneal tenography, FE: fluoroscopic evaluation, ROC: receiver operating characteristic curves, AUC: area under the curve.

**(E) Summary of Explorations on Minimal Detectable Change for the ADT**

| **Origin** | **Gauge** | **Performance** | **MDC *** | |
| --- | --- | --- | --- | --- |
|  |  |  | ***Based on***  ***the intra-rater reliability*** | ***Based on***  ***the inter-rater reliability*** |
| Kataoka  2022 | Electromagnetic  Sensor | Manual test | Rater 1: 0.610 mm #  Rater 2: 0.748 mm #  Rater 3: 0.748 mm # |  |
| Parasher  2012 | Plastic  Goniometer | Manual test | Rater 1: 1.995 mm  Rater 2: 1.663 mm | Between the 2 raters: 6.291 mm |
| Wilkin  2012 | 8-point  Likert scale  (Key-anchors:  -2 very stiff,  0 normal stability,  5 hypermobility) | Manual test |  | In supine:  Between the 3 raters: 3.076 units  (1 novice and 2 experienced)  Between the 2 raters: 3.298 units  (Experienced)  In crook lying:  Between the 3 raters: 3.852 units  (1 novice and 2 experienced)  Between the 2 raters: 4.684 units  (Experienced) |
| Docherty  2009 | Digital  Arthrometer | Instrumented test | Rater 1: 6.153 mm  (on 2 separate days) | Between the 2 raters: 4.462 mm |

MDC: minimal detectable change.

*: It was calculated using the formula: $MDC=SEM\times\sqrt{2}\times1.96$ .

#: The values marked indicate MDCs obtained by first converting SD to SEM before calculation ( $SEM=SD\times\sqrt{1-ICC}$ ).

**(F) Study limitations**

One of the key limitations of this review lies in the variability and inconsistency found across the included studies. Many of the studies did not provide detailed information on the qualifications, backgrounds, and experiences of the examiners conducting the drawer tests, which could impact the reliability of the results. Additionally, the studies often lacked specific details regarding the interval times between tests, which is a crucial factor in determining the consistency of the measurements. This omission has led to lower quality ratings for some of the studies included in the review. Finally, the arthrometers used across studies may not be the same, and the force parameters applied may differ, which could influence the comparability of results between studies.

Another significant limitation is the heterogeneity of the study populations. The demographics and injury diagnoses of the subjects varied widely across the studies, with some even including cadaveric specimens. This variability in study populations introduces potential biases in the conclusions drawn from these studies, as the results may not be generalizable to all patient groups. Furthermore, the reference standards used for comparison in different studies varied in terms of equipment and parameters, which could affect the diagnostic accuracy of the ADT. The lack of standardization in these reference standards adds another layer of uncertainty to the findings.

Lastly, the differences in the experimental settings across studies also pose a limitation. Some studies were conducted in hospitals with patients, others in laboratories with subjects, and still others in educational settings with students. These varied environments may influence the examiners’ focus and the subjectivity of their evaluations. The level of care and attention given by the examiner when performing the ADT in different contexts could lead to variability in the results, potentially impacting the overall assessment of the test’s accuracy and reliability.
